# Anti-Cancer Effects of Pristimerin and the Mechanisms: A Critical Review

**DOI:** 10.3389/fphar.2019.00746

**Published:** 2019-07-12

**Authors:** Jia-jun Li, Yan-yan Yan, Hong-mei Sun, Yun Liu, Chao-yue Su, Hu-biao Chen, Jian-ye Zhang

**Affiliations:** ^1^Guangdong Provincial Key Laboratory of Molecular Target & Clinical Pharmacology, School of Pharmaceutical Sciences and the Fifth Affiliated Hospital, Guangzhou Medical University, Guangzhou, China; ^2^Institute of Respiratory and Occupational Diseases, Collaborative Innovation Center for Cancer, Medical College, Shanxi Datong University, Datong, China; ^3^School of Chinese Medicine, Hong Kong Baptist University, Hong Kong, China; ^4^Infinitus (China) Company Ltd., Jiangmen, China

**Keywords:** pristimerin, anti-cancer, mechanism, molecular target, pharmaceutical effect, apoptosis, autophagy

## Abstract

As a quinonemethide triterpenoid extracted from species of the *Celastraceae* and *Hippocrateaceae*, pristimerin has been shown potent anti-cancer effects. Specifically, it was found that pristimerin can affect many tumor-related processes, such as apoptosis, autophagy, migration and invasion, vasculogenesis, and drug resistance. Various molecular targets or signaling pathways are also involved, such as cyclins, reactive oxygen species (ROS), microRNA, nuclear factor kappa B (NF-κB), mitogen-activated protein kinase (MAPK), and PI3K/AKT/mammalian target of rapamycin (mTOR) pathways. In this review, we will focus on the research about pristimerin-induced anti-cancer activities to achieve a deeper understanding of the targets and mechanisms, which offer evidences suggesting that pristimerin can be a potent anti-cancer drug.

## Introduction

**Figure 1:**
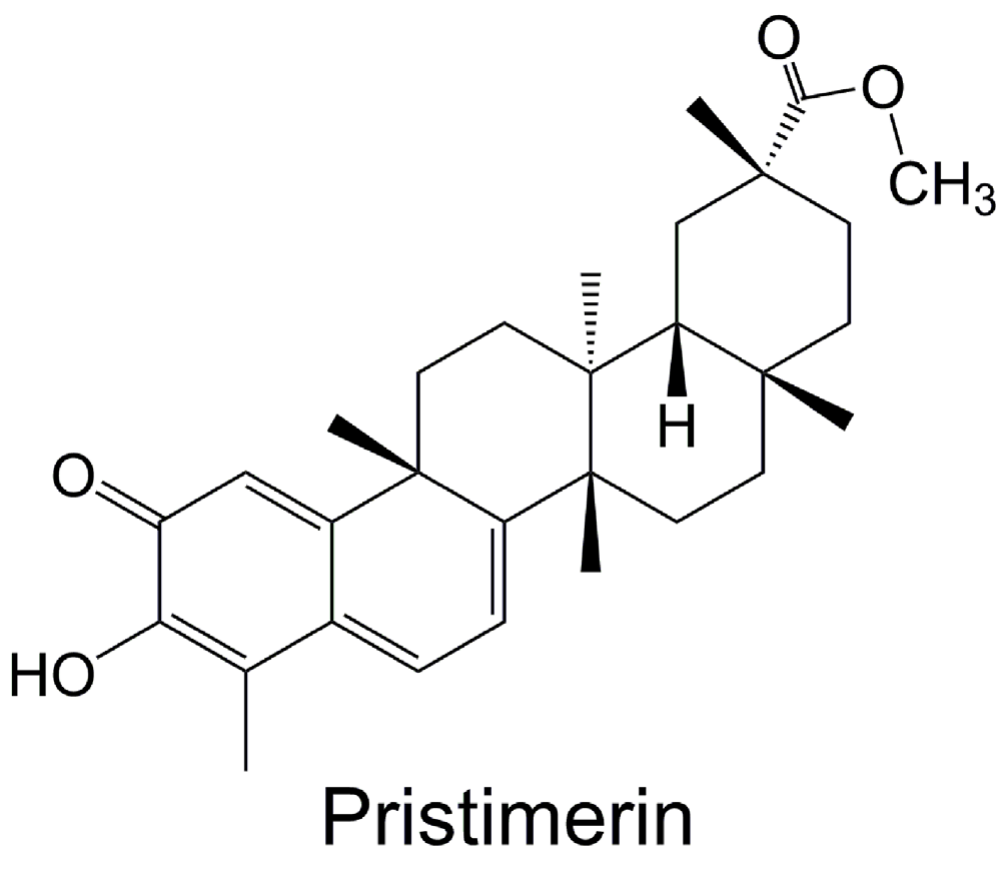
Chemical structure of pristimerin.

In recent years, natural compound has received more and more attention for use in treating human diseases and conditions, due to their long history of use and various pharmacological therapeutic effects (Tao et al., 2015; Zhang et al., 2015; Peng et al., 2016; Zhang et al., 2016; Lin et al., 2017), especially their relative safety (fewer and less severe side effects) than chemical drugs. Naturally occurring triterpenoid can be used as anti-cancer, anti-inflammatory, anti-malarial, and insecticidal agent (Deeb et al., 2012; Larsen et al., 2012; Kim et al., 2013; Deeb et al., 2014a). It has been proven that some natural or synthetic triterpenoids have promising clinical potential, exhibiting both therapeutic and chemopreventive activities for cancer (Salminen et al., 2008; Alessia et al., 2009; Ke et al., 2016). Pristimerin (20α-3-hydroxy-2-oxo-24-nor-friedela-1-10,3,5,7-tetraen-carboxylic acid-29-methylester, molecular formula: C_30_H_40_O_4_) (**Figure 1**), a methyl ester of celastrol, is a quinonemethide triterpenoid which has been extracted from a variety of species of the *Celastraceae* and *Hippocrateaceae* families, such as *Hippocratea excels* (Mena-Rejon et al., 2007),* Maytenus heterophylla* (Murayama et al., 2007), and *Celastrus aculeatus* Merr. (Tang et al., 2014). Pristimerin was first isolated in 1951 from *Pristimerae indica* and *P. grahami* and was first identified in 1954 to confirm its molecular structure (Kulkarni and Shah, 1954). Pristimerin has displayed different pharmacological effects, such as anti-cancer, anti-oxidant, anti-inflammatory, anti-bacterial, anti-malarial, and insecticidal activities (Figueiredo and Sequin, 1998; Avilla et al., 2000; Haroldo Jeller et al., 2004; Lopez et al., 2011; Kim et al., 2013; Wu et al., 2019). As such, it is being developed as a potential anti-cancer drug (Yousef et al., 2017). Here, we present and discuss current research findings with regard to pristimerin emphasis on the anti-cancer effect.

## Pristimerin: Broad-Spectrum Anti-Cancer Effect

Cancer is a complicated disease, which starts with a normal change through the activation of proto-oncogenes or the suppression of tumor suppressor genes (Elmore, 2007). These alterations result in diversed and interactive changes at the level of cellular processes which are involved in the regulation of proliferation, differentiation, apoptosis, migration, and tissue homeostasis. Finally, biological properties for cancer cells are acquired, including infinite proliferation potential, independent exogenous growth factors, and resistance to death signals (Brattain et al., 1994; Dent and Aranda-Anzaldo, 2019; Petho et al., 2019; Shen et al., 2019).

Pristimerin exerts its effects influencing a series of biological properties of cancer cells. Recent studies on a wide range of cancer cell lines of different origins, such as oral cancer (Wu et al., 2019), colorectal cancer (Yousef et al., 2018), glioma (Yan et al., 2013), leukemia (Lu et al., 2010), breast cancer (Xie et al., 2016), lung cancer (Zhang et al., 2019), and prostate cancer (Liu et al., 2013), and also in cancer stem cells (Cevatemre et al., 2018). These results have proved that pristimerin possesses strong anti-proliferative activities with involvement of mitochondrial apoptosis, autophagy, and inhibition of nuclear factor kappa B (NF-κB), Akt (protein kinase B, PKB) and mitogen-activated protein kinase (MAPK) (Guo et al., 2013; Liu et al., 2013; Yan et al., 2013; Gao et al., 2014; Deeb et al., 2015).

In view of the potent anti-cancer effect in a broad spectrum (cancer cell lines and molecular targets), it possesses a great potential for pristimerin to develop as a multiple-target anti-cancer drug.

## Pristimerin: Anti-Cancer Activities

### Growth Inhibition

Pristimerin induces a potent effect of growth inhibition within wide range types of human tumors; the cytotoxicity of pristimerin in different cancer cell lines is summarized in **Table 1**.

**Table 1:** The cytotoxicity dosage of pristimerin in different cancer cell lines

Cancer type	Time	Toxic dosage (IC_50_ value or inhibition rate)	References
Prostate cancer	72 h	1.25 µM caused 55% LNCaP cell death	(Liu et al., 2013)
		1.25 µM caused 47% PC-3 cell death	
Breast cancer	24 h	2.40 µM IC_50_ against SKBR3	(Lee et al., 2013)
Colorectal cancer	72 h	1.11 µM IC_50_ against HCT-116	(Yousef et al., 2018)
	48 h	1.22 µM IC_50_ against HCT-116	(Yousef et al., 2016a)
		1.04 µM IC_50_ against SW-620	
		0.84 µM IC_50_ against COLO-205	
Hepatocellular carcinoma	72 h	1.44 µM IC_50_ against HepG2	(Guo et al., 2013)
		1.70 µM IC_50_ µM against HepG2	(Wei et al., 2014)
		0.68 µM IC_50_ µM against Huh7	
		0.85 µM IC_50_ µM against Hep3B	
Pancreatic cancer	24 h	0.66 µM, 0.97 µM, 0.13 µM, IC_50_ against BxPC-3, PANC-1, and AsPC-1, respectively	(Wang et al., 2012)
	48 h	0.28 µM, 0.34 µM, and 0.38 µM IC_50_ against BxPC-3, PANC-1, and AsPC-1, respectively	
	72 h	0.19 µM, 0.26 µM and 0.30 µM IC_50_ against BxPC-3, PANC-1, and AsPC-1, respectively	
Glioma	6 h	4.5 µM IC_50_ against U251	(Zhao et al., 2016)
		5.0 µM IC_50_ against U87	
Leukemia	72 h	0.61 µM IC_50_ against HL-60	(Costa et al., 2008)
		1.49 µM IC_50_ against K562	
	72 h	199 nM IC_50_ against KBM5	(Lu et al., 2010)
		135 nM IC_50_ against KBM5-T315I	
		450 nM IC_50_ against K562	
Ovarian carcinoma	72 h	1.25 µM caused 44% OVCAR-5 cell death	(Gao et al., 2014)
		1.25 µM caused 28% MDAH-2774 cell death	
		2.5 µM caused 36% SK-OV-3 cell death	
		2.5 µM caused 27% OVCAR-3 cell death	
Osteosarcoma	24 h	0.80 µM IC_50_ against MNNG	(Mori et al., 2017)
		0.54 µM IC_50_ against 143B	
	48 h	0.39 µM IC_50_ against MNNG	
		0.31 µM IC_50_ against 143B	
	72 h	0.32 µM IC_50_ against MNNG	
		0.29 µM IC_50_ against 143B	
Oral cancer	72 h	0.54 µM IC_50_ against KB	(Yan et al., 2017)
		0.52 µM IC_50_ against KBv200	
		0.70 µM IC_50_ against CAL-27	(Wu et al., 2019)
		0.73 µM IC_50_ against SCC-25	
ESCC	72 h	1.98 µM IC_50_ against EC9706	(Tu et al., 2018)
		1.76 µM IC_50_ against EC109	
		1.13 µM IC_50_ against KYSE30	

ESCC, esophageal squamous cell carcinoma.

### Apoptosis Induction

Apoptosis is a kind of programmed cell death, whose activation is regulated by a series of genes, in the purpose of eliminating redundant, damaged, even infected cells to maintain homeostasis (Ke et al., 2016). Anti-cancer agents killing tumor cells by the induction of apoptosis is generally studied (Wu et al., 2017; Xiao et al., 2018; Qi et al., 2019). Two main subtypes of apoptosis have been divided into the intrinsic mitochondrial pathway and the extrinsic death receptor pathway (Elmore, 2007).

In the mitochondrial pathway, Bcl-2 family members converge on mitochondria (Kale et al., 2018), regulating release of various mitochondrial components to form the apoptosome (Dorstyn et al., 2018), such as cytochrome *c* associated with Apaf-1 and procaspase-9 (Estaquier et al., 2012). In the death receptor pathway, stimulation of death receptors, including Fas and tumor necrosis factor (TNF) receptor-1, results in the assembly of death-inducing signaling complex, containing the adapter protein (Gupta, 2001), Fas-associated death domain, and initiator caspases, such as caspase-8 (Pecina-Slaus, 2009).

Pristimerin-induced apoptotic effects were mainly due to mitochondrial dysfunction, activation of both extrinsic and intrinsic caspases, and cleavage of poly ADP-ribose polymerase (PARP). It has been reported that pristimerin can induce caspase-dependent apoptosis in human glioma cancer cells (Yan et al., 2013), pancreatic cancer cells (Deeb et al., 2014b), and hepatoma cancer cells (Gao et al., 2014). Pristimerin-induced inhibition of Bcl-2 (as well as Bcl-2 mRNA) is sufficient to promote mitochondrial permeability transition and release of cytochrome *c* mediated by Bax and Bak without the inhibition of Bcl-xL in pancreatic cancer cells (Deeb et al., 2014b). On the other hand, caspase inhibitor failed to antagonize the effects of pristimerin, indicating that the lethal effect of pristimerin may not be caspase-dependent in human glioma U251 and U87 cells (Zhao et al., 2016).

The apoptotic effect of pristimerin is related to Bcl-2, and it mediates down-regulation of Bcl-2 through reactive oxygen species (ROS)-dependent ubiquitin-proteasomal degradation pathway in human prostate cancer LNCaP and PC-3 cells (Liu et al., 2013). ROS-induced apoptosis by pritimerin was also reported in hepatocellular carcinoma HepG2 cells, involving EGFR and Akt proteins (Guo et al., 2013). In colorectal carcinoma cells, the associated induction of JNK activation and MMP loss was observed (Yousef et al., 2016b), similar with the results in cervical cancer cells (Byun et al., 2009).

In human colon cancer cells, pristimerin caused cell cycle arrest and apoptosis through cyclin-CDK, mitochondrial dysfunction, and caspase-dependent mechanisms. Besides, the inhibition of DNA synthesis in HL-60 was also associated with pristimerin-induced apoptosis (Costa et al., 2008).

Pristimerin-induced apoptosis could be mediated by microRNA (miRNA). miRNAs exert a post-transcriptional gene silencing effect through binding to target mRNA and endonucleolytic cleavage of the mRNA by protein argonaute-2 (AGO2) (Kobayashi and Tomari, 2016). It was reported that pristimerin induced apoptosis through inhibiting AGO2 and PTPN1 expression *via* miR-542-5p in glioma cancer cells U373 (Li et al., 2019). Synergization with cisplatin, pristimerin led to apoptosis *via* inhibiting the miR-23a, regulating PTEN/Akt signaling-related PTEN and the phosphorylation of Akt and GSK3β in lung carcinoma NCI-H446 and A549 cells (Zhang et al., 2019).

### Autophagy Induction

As another programmed necrosis, autophagy is a homeostatic cellular self-digestive process. Autophagy triggered by various cellular stress plays vital role in cell death, providing novel target for developing anti-cancer drug (Mizushima et al., 2008; Ravanan et al., 2017). LC3-II promotes the expansion and maturation of autophagy, which is considered as signal of autophagy activation. Pristimerin-induced autophagy was reported in human breast cancer MDA-MB-231 (Cevatemre et al., 2018; Lee et al., 2018) and MCF-7 cells (Cevatemre et al., 2018). As evidenced by the increase of p62 and LC3-II with an unfolded protein response (UPR), pristimerin induced an incompleted autophagy through Wnt signaling. Although endoplasmic reticulum (ER) stress is also a trigger of autophagy (Smith and Wilkinson, 2017), it was not concluded whether the observed ER stress by pristimerin induced autophagy (Cevatemre et al., 2018). Additionally, a combination treatment of pristimerin and paclitaxel strengthened the extracellular signal-related kinase (ERK)-dependent autophagic cell death, with increase of p62 degradation and beclin1 expression (Lee et al., 2018).

On the contrary, pristimerin suppressed autophagy, downregulating LC3BII and beclin1 to sensitize the apoptosis caused by cisplatin in lung carcinoma A549 and NCI-H446 cells (Zhang et al., 2019).

### Inhibition of Metastasis, Migration, Invasion, Angiogensis, and Cancer Stem Cell

The cancer metastases include a series of process, such as the completion of a complex succession of cell-biological event, cancer cell invasion, migration, and forming metastatic colonization in clinic (Valastyan and Weinberg, 2011). Pristimerin was reported to inhibit migration and invasion *via* targeting G protein signaling 4 (RGS4) in breast cancer MDA-MB-231 cells (Mu et al., 2012a) and HER2 in human breast carcinoma SKBR3 cells (Lee et al., 2013). Furthermore, mammalian target of rapamycin (mTOR) may be associated with its upstream Akt in pristimerin-induced inhibition of migration and invasion in colorectal cancer HCT-116 cells (Yousef et al., 2016b). Pristimerin suppressed the invasion of human prostate cancer PC-3 cells through inhibition of epithelial-to-mesenchymal transition (EMT), which was confirmed by the EMT-related markers (Chaffer et al., 2016), including N-cadherin, fibronectin, vimentin and ZEB1 (Zuo et al., 2015). MMP2 and MMP9, which are important proteins regulating invasion and metastasis, were decreased by pristimerin in esophageal cancer EC9706 and EC109 cells in a dose-dependent manner, resulting in inhibition of migration and invasion (Tu et al., 2018).

To supply nutrients and clear metabolic wastes, novel capillary blood vessels grow from pre-existing vasculature, which is called angiogenesis. However, aberrant angiogenesis plays a key role in cancer development (Valastyan and Weinberg, 2011). Thus, anti-angiogenic therapy is promising and under development (Li et al., 2018). Pristimerin was reported to *in vivo* inhibit the neovascularization of chicken chorioallantoic membrane (CAM) and vessel *ex vivo* sprout in rat aortic ring assay, through a vascular endothelial growth factor (VEGF)-dependent mechanism (Mu et al., 2012b). Also, the decreased-VEGF by pristimerin was reported through the inhibition of HIF-1α *via* the SPHK-1 signaling pathway in hypoxic prostate cancer PC-3 cells (Lee et al., 2016). In addition, pristimerin-induced cancer stem cell toxicity was observed in breast cancer stem cells (Cevatemre et al., 2018) and esophageal squamous cell carcinoma (ESCC) (Tu et al., 2018).

### Reversal of Drug Resistance

Multi-drug resistance (MDR) is defined as the resistance of cancer cells not limited to a specific chemotherapeutic drug through different structures and mechanisms of action (Wu et al., 2014). ABCB1 (P-glycoprotein, Pgp) is recognized as putative drug transporter, which is encoded by the ABCB1 gene, one of (ATP)-binding cassette (ABC) transporter family (Dewanjee et al., 2017). Pristimerin may overcome ABCB1-mediated chemotherapeutic drug resistance through disturbing the stability of ABCB1 independent of its mRNA expression in human oral epidermoid carcinoma cells KBv200 (Yan et al., 2017). In addition, with inhibition of NF-κB and Bcr-Abl, pristimerin is effective *in vitro* and *in vivo* against imatinib-resistant chronic myelogenous leukemia cells (Lu et al., 2010). Additionally, Akt signaling was related to the reversal of MDR in multidrug-resistant MCF-7/ADR breast cancer cells (Xie et al., 2016).

### Synergization With Chemotherapeutic Drugs

Drug combination for cancer treatment has been well established to strengthen the anti-tumor action in varied aspects (Ho and Cheung, 2014; Andre et al., 2018), including therapeutic drug combination with natural product (Efferth, 2017; Sanchez et al., 2019). Pristimerin was reported to synergize with paclitaxel in human breast cancer cells (Lee et al., 2018), with 5-fluorouracil (5-FU) in esophageal ESCC (Tu et al., 2018). In cervical cancer cells, combination with taxol could induce cell death through ROS-mediated mitochondrial dysfunction (Eum et al., 2011). In NCI-H446 and A549 lung carcinoma cells, combination with cisplatin could induce cell apoptosis through inhibiting the miRNA-23a and Akt/GSK3β signaling pathway (Zhang et al., 2019). In pancreatic cancer cells, pristimerin could potentiate the cytotoxic effect of gemcitabine with the possible mechanism being the inhibition of gemcitabine-induced NF-κB activation (Wang et al., 2012).

### *In Vivo* Anti-Tumor Activities

Pristimerin was widely reported its *in vivo* anti-tumor activities, which is summarized in **Table 2**.

**Table 2:** *In vivo* anti-tumor activities of pristimerin.

Models	Dose and administration	Activities	Mechanisms	References
Human breast tumor xenograft model	3 mg/kg/2 days, s.c.	Reduced both tumor volume and tumor weight, inhibited tumor angiogenesis.	Associated with decreased secretion of proangiogenic molecules (VEGF)	(Mu et al., 2012b)
Human breast tumor xenograft model	1 mg/kg/2 days, s.c.	Inhibited the growth of implanted tumors, inhibited the invasiveness	—	(Mu et al., 2012a)
Orthotopic HCC patient-derived xenograft model	1 mg/kg/3 times/week, i.v.	Caused significant reductions in tumor volumes of xenografts	Disrupt HSP90 and CDC37 interaction, inhibit Raf/MEK/ERK and PI3K/AKT/mTOR pathways	(Wei et al., 2014)
Intra-tibial injection model	7.5 × 10^3^ cells/µl 1.6 µM pristimerin pre-treated 24 h PC-3 cells	Inhibited the bone destruction by the invasion of the tumor, reduced the tumorigenic potential of bone metastasis	—	(Huang et al., 2015)
Human glioma xenograft model	1 and 3 mg/kg/2 days, s.c.	Inhibited glioma volume and weight *in vivo* in a dose-dependent manner	Up-regulated JNK level the phosphorylated JNK, upregulated the nuclear AIF and the ratio of Bax/Bcl-2	(Zhao et al., 2016)
AOM/DSS model of colitis-associated colorectal carcinogenesis	fed with 1 to 5 ppm pristimerin	Reduced tumor burden	—	(Park and Kim, 2018)
Human ESCC xenograft model	1 mg/kg/2 days, i.t.	Inhibited the growth and weight of tumor, suppressed proliferation	—	(Tu et al., 2018)
Human colorectal cancer xenograft model	1 mg/kg/2 days, i.p.	Inhibited tumor growth	Mainly through suppressing NF-кB activity and p65 phosphorylation	(Yousef et al., 2018)
Human lung tumors xenograft model	0.8 mg/kg pristimerin and 2 mg/kg cisplatin, s.c.	Enhanced the effect of cisplatin to decrease tumor volumes and weights	Inhibited the phosphorylation of Akt and GSK3β	(Zhang et al., 2019)
Human osteosarcoma xenograft model	1 mg/kg/2 days, i.p.	Reduced both tumor volume and tumor weight	—	(Mori et al., 2017)
Human colorectal cancer xenograft model	1 mg/kg/2 days, i.p.	Inhibited the growth of implanted tumors	Induced apoptosis through an increment in cleaved caspase-3	(Yousef et al., 2016b)
Human myeloma xenograft model	2.5 mg/kg per day, s.c.	Inhibited growth of human myeloma xenograft, diminished toxicity in a liposomal dose	—	(Tiedemann et al., 2009)
Human breast cancer xenograft model	1 mg/kg for 2 days, i.p.	Decreased tumor size and weights, slightly reduced toxicity and behavioral changes in an E/T80/WFI carrier compared to D/PBS.	—	(Cevatemre et al., 2018)

*s.c. represents subcutaneously, i.v. for intravenously, i.t. for intratumorly, i.p. for intraperitoneally, and ppm for parts per million, respectively. ERK, extracellular signal-related kinase.

## Pristimerin in Tumors: Targets and Pathways

### Proteasome

As another important mechanism of maintaining homeostasis, proteasome-mediated degradation is associated with essential cellular processes, regulating the vast majority of cellular proteins (Livneh et al., 2016). Consistent with triterpenoids being reported to target proteasome (Chintharlapalli et al., 2007; Tiedemann et al., 2009), pristimerin also showed a potent activity to inhibit proteasome activity in prostate cancer cells (Yang et al., 2010; Liu et al., 2013; Liu et al., 2014), breast cancer cells (Mu et al., 2012a), cervical carcinoma cells (Eum et al., 2011), and myeloma cells (Tiedemann et al., 2009).

The β subunits of proteasome contain active protease sites with different peptidase activities, including caspase-like or peptidyl-glutamyl peptide-hydrolyzing-like (β1), trypsin-like post basic (β2), and chymotrypsin-like (β5) activities (Mayor et al., 2016). Pristimerin was associated with the N-terminal threonine of the β5 subunit through its conjugated ketone carbon C_6_, exerting a chymotrypsin-like activity (Yang et al., 2010), which is also associated with RGS4 (Mu et al., 2012a).

Pristimerin can inhibit Bcl-2, finally induced mitochondrial cell death *via* an ROS-dependent ubiquitin-proteasomal degradation pathway (Liu et al., 2013). Pristimerin combination with taxol caused mitochondrial apoptosis due to ROS generation and direct proteasome inhibition (Eum et al., 2011). In addition, pristimerin-induced inhibition of proteosome and IKK phosphorylation of IκB together led to UPR and suppression of NF-κB activity and cyclin D2 expression in myeloma cells H929 and U266 (Tiedemann et al., 2009).

### Telomerase

Telomere is a ribonucleoprotein complex located in the end of chromosomes, maintaining telomere length homeostasis to keep chromosomal stability (Wang and Feigon, 2017). Due to the differences in telomere homeostasis between cancer and normal cells, targeting telomerase may be a promising approach to find effective and safe anti-cancer treatments (Armstrong and Tomita, 2017).

Pristimerin can inhibit telomerase activity in human prostate cancer LNCaP and PC-3 cells (Liu et al., 2015). The mechanism is related to inhibition of human telomerase reverse transcriptase (hTERT) and its mRNA expression, which codes the catalytic subunit of the telomerase. At the same time, knocking-down of hTERT strengthened the effects of pristimerin. Furthermore, hTERT regulatory proteins c-Myc, Sp1, p-STAT3, and p-Akt were inhibited in a dose-dependent manner (Liu et al., 2015).

### MAPK Pathway

The generic MAPK signaling pathway is co-regulated by four different cascades including extracellular signal-related kinases (ERK1/2), Jun amino-terminal kinases (JNK1/2/3), p38-MAPK, and ERK5 (Sun et al., 2015). MAPK/ERK pathway regulates the cell proliferation (Sun et al., 2017), differentiation (Wang et al., 2017), migration (Tao et al., 2018) and apoptosis (Wang and Zhu, 2018).

Pristimerin-induced autophagy was reported *via* ERK1/2 in human breast cancer cells when combination with paclitaxel (Lee et al., 2018). ERK1/2 may be involved in pristimerin-induced intrinsic apoptosis in human oral epidermoid carcinoma cells (Yan et al., 2017) and in human glioma cells (Yan et al., 2013). Both JNK and PARP-1 *via* ROS pathway are essentially required for the pristimerin-induced intrinsic apoptosis in human cervical cancer cells (Byun et al., 2009). In addition, ERK1/2 suppression occurred in VEGF-induced capillary-like structure formation of human umbilical vascular endothelial cells (HUVECs) (Mu et al., 2012b). These activities were accompanied with Akt inhibition (Mu et al., 2012b; Yan et al., 2017; Lee et al., 2018).

### PI3K/AKT/mTOR Pathway

The phosphatidylinositol 3-kinase (PI3K)/AKT/mTOR pathway cascade containing PI3K, AKT, and mTOR is the most frequently altered pathway in human for cancer development, such as cell cycle, cell survival, metabolism, motility, angiogenesis, chemoresistance, and genomic instability (Mabuchi et al., 2015).

Pristimerin showed a potent apoptosis-inducing anti-proliferative activity in human osteosarcoma cells (Mori et al., 2017) by PI3K/AKT/mTOR pathway. The pristimerin-induced ROS-dependent mitochondrial cell apoptosis was also associated with the inhibition of EGFR and Akt in human glioma cells (Yan et al., 2013). It was confirmed that PI3K/AKT/mTOR pathway-activated activities were accompanied by the downstream Foxo-3α, cyclin D1 and Bcl-XL (Akt), p-S6K1, and p-4E-BP1 (mTOR) as well as p21, p27, and PKCε in human ovarian cancer cells (Deeb et al., 2014b; Gao et al., 2014; Park and Kim, 2018). Furthermore, downstream Bad and Bcl-xL pointed to drug resistance in MCF-7/ADR human breast cancer cells (Xie et al., 2016). In addition, pristimerin suppressed angiogenesis through VEGF-induced Akt, ERK1/2, mTOR, and ribosomal protein S6 kinase (Mu et al., 2012b).

### NF-κB Pathway

NF-κB family transcription factors are crucial regulators of cell survival and inflammatory processes (Napetschnig and Wu, 2013). The inactive NF-κBs are isolated from nucleus by inhibitor of NF-κB (IκB) proteins. When activated IKK (IκB kinase) makes a proteasomal degradation of IκB, the subsequent process will occur, including the release of NF-κB, translocation of NF-κB nuclear and activation of gene transcription. NF-κB can be activated by both intracellular and extracellular stimuli, including cytokines (TNF-α, IL-1β), bacterial, and viral products (LPS) (Xia et al., 2014).

NF-κB-regulated anti-apoptotic Bcl-2, Bcl-xL, c-IAPl, and surviving in human ovarian carcinoma cells (Gao et al., 2014), Cox-2 and VEGF in human pancreatic cancer cells (Deeb et al., 2014b). NF-κB pathway may link anti-tumor activity of pristimerin and its anti-inflammatory properties (Park and Kim, 2018). Pristimerin suppressed the translocation of NF-κB nuclear; however, there was no change of the total NF-κB protein in pancreatic cancer (Wang et al., 2012). In contrast, pristimerin inhibited both genetic expression and activation of NF-кB protein with suppression of p65 mRNA in human colorectal cancer cells (Yousef et al., 2018). TNFα-induced NF-κB activation was observed by the downstream MMP9, cyclin D1, and c-Myc in ESCC cells (Tu et al., 2018). When combined with pristimerin, the inactivation of Bcr-Abl by imatinib did not interfere with the TNFα-induced NF-κB activation, which implicated that NF-κB inactivation and Bcr-Abl inhibition may be parallel mechanisms of pristimerin-induced activity in human chronic myelogenous leukemia cells (Lu et al., 2010). G1 phase arrest was also associated with NF-κB pathway in human pancreatic cancer cells (Wang et al., 2012), as well as proteosome in human myeloma cells (Tiedemann et al., 2009). Moreover, pristimerin inhibited expression of miR-542-5p targeting PTPN1, which encodes protein tyrosine phosphatase 1B (PTP1B) related to NF-κB pathway (Li et al., 2019).

### Wnt/β-Catenin Pathway

Wnt proteins are key mediators in a series of important cellular process. The abnormal activation of Wnt/β-catenin pathway can cause a wide range of diseases including cancers (Krishnamurthy and Kurzrock, 2018; Pedone and Marucci, 2019). Pristimerin was reported to suppress Wnt/β-catenin pathway through targeting and inhibiting the expression of LRP6 and its phosphorylation, which may contribute to autophagy in human breast cancer MCF-7 cells (Cevatemre et al., 2018).

## Conclusions and Perspective

Plants, particularly medicinal herbs, have become increasingly popular due to their potent therapeutic effects. Pristimerin, a quininemethide triterpenoid compound isolated from species of the *Celastraceae* and *Hippocrateaceae* families, has displayed biological and pharmacological activities, particularly inhibiting cancer. This review summarizes the reported results on anti-cancer activities and related mechanisms of pristimerin.

**Figure 2:**
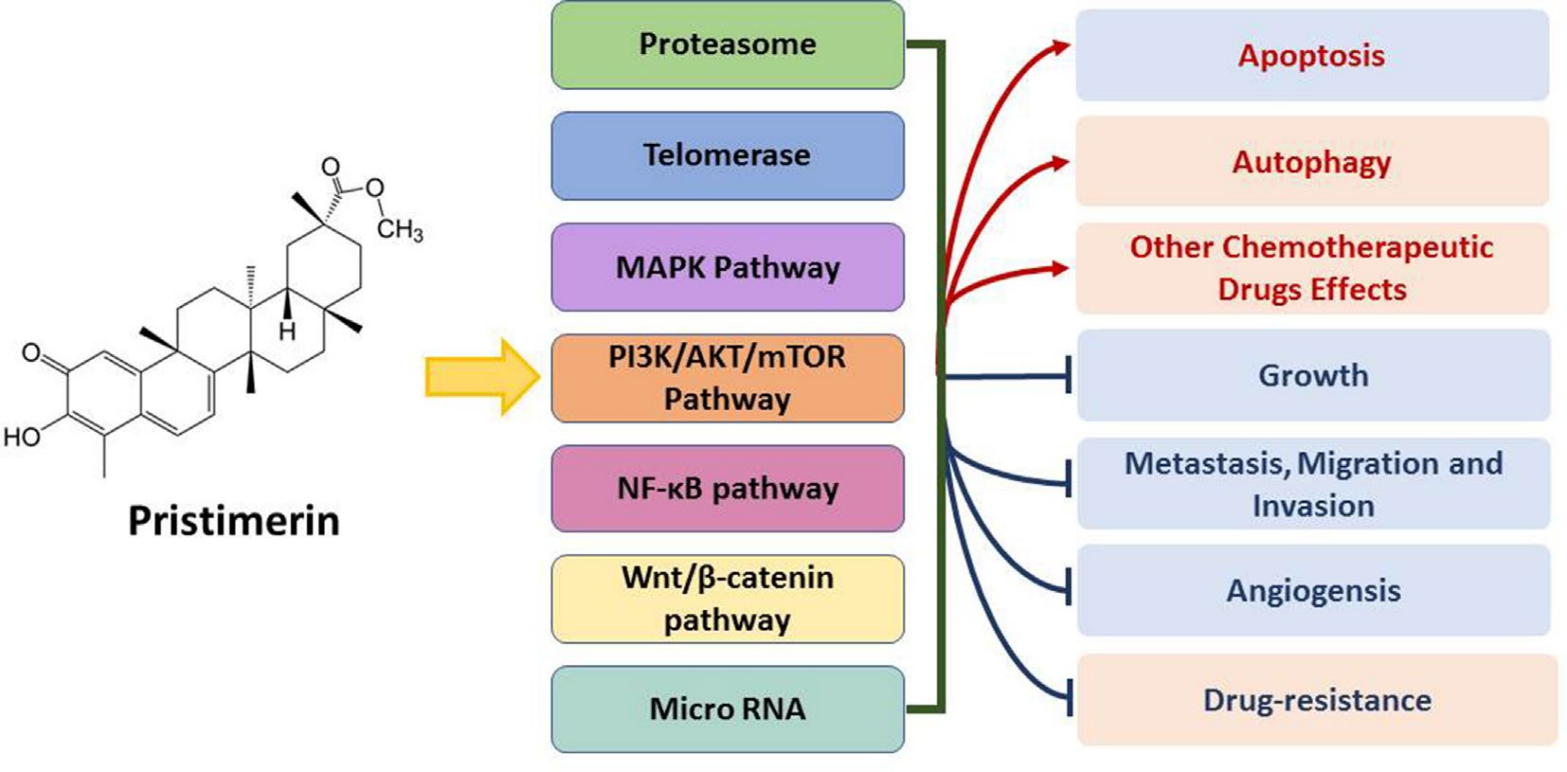
Brief summary of anti-cancer mechanisms and activities of pristimerin.

Pristimerin has shown anti-cancer potency *in vivo* (**Table 2**) and *in vitro* (**Table 3**) *via* specific mechanisms (**Figure 2**). Like many other chemotherapeutic drugs, pristimerin exerts cytotoxicity largely related to apoptosis, while the mechanism of autophagy is merely reported. The cross-talk of apoptosis and autophagy mediated by pristimerin is still remained to be explored. So far, the mechanism study of pristimerin has little reported on lung cancer, epigenetic regulation, and combination with immunotherapy. Furthermore, pristimerin has been reported to have poor selective toxicity in some cancer cells or compared with its derivatives (Costa et al., 2008; Wei et al., 2014). Comprehensive evaluation of pristimerin toxicity is yet to be carried out (as well as clinical trials). In summary, pristimerin possesses potent anti-cancer effect and further study will bring about novel drug development based on pristimerin.

**Table 3:** Anti-cancer mechanisms of pristimerin in different cell lines.

Cancer type	Cell lines	Mechanisms	References
Prostate cancer	PC-3	Inhibited HIF-1α accumulation by inhibiting SPHK-1	
		Inhibited CD133 and CD44 protein expression, reduced VEGF	(Huang et al., 2015)
	LNCaP and PC-3	Down-regulated Bcl-2 through an ROS-dependent ubiquitin-proteasomal degradation pathway	(Liu et al., 2013)
		Prevented survivin *via* the ubiquitin-proteasome pathway	(Liu et al., 2014)
		Inhibited hTERT expression *via* the inhibition of SP1, c-Myc, STAT3, and B/Akt	(Liu et al., 2015)
Breast cancer	SKBR3	Down-regulated HER2, decreased fatty acid synthase	(Lee et al., 2013)
	MDA-MB-231	Suppressed proteasomal activity *via* increasing the levels of RGS4	(Mu et al., 2012a)
		Suppressed the LC3-II levels of this on ERK signaling when combination with paclitaxel	(Lee et al., 2018)
Colorectal cancer	HCT-116	Inhibited the AKT/FOXO3a pathway *via* decreasing cyclinD1 and Bcl-XL, increased the expression of p21 and p27	(Park and Kim, 2018)
	HCT-116	Inhibited activated NF-кB, TNFα, and activated LPS-induced NF-кB signaling pathway	(Yousef et al., 2018)
	HCT-116, COLO-205, and SW-620	Inhibited of phosphorylated EGFR and HER2 expression, caused inhibition of related downstream kinases.	(Yousef et al., 2016a)
Hepatocellular carcinoma	HepG2	Generated ROS, induced release of cytochrome *c*, and down-regulated EGFR protein	(Guo et al., 2013)
		Disrupted HSP90/CDC37 interaction, degraded and inhibited phosphorylation of protein kinases in the Raf/MEK/ERK and PI3K/AKT/mTOR signaling pathways	(Wei et al., 2014)
Pancreatic cancer	BxPC-3, PANC-1, and AsPC-1	Inhibited of the translocation and DNA-binding activity of NF-κB	(Wang et al., 2012)
	MiaPaCa-2 and Panc-1	Inhibited of hTERT *via* suppressing the transcription factors Sp1, c-Myc, and NF-κB	(Deeb et al., 2015)
Glioma	U87	Activated of JNK through overproduction of ROS	(Zhao et al., 2016)
	U373	Targeting AGO2 and PTPN1 expression *via* miR-542-5p	(Li et al., 2019)
Myeloma	H929 and U266	Both inhibited IKK phosphorylation of IκB and proteosome, causing unfolded protein response and suppressing NF-κB activity and cyclin D expression	(Tiedemann et al., 2009)
Cervical cancer	HeLa	Activated ROS-dependent JNK, Bax, and PARP-1	(Eum et al., 2011)
Leukemia	HL-60	Interfered DNA synthesis	(Costa et al., 2008)
	KBM5 and KBM5-T3151	Depleted Bcr-Abl, activated TAK1TIKK and IKKTIκBα in NF-κB signaling parallel but independent	(Lu et al., 2010)
Ovarian carcinoma	OVCAR-5, MDAH-2774, OVCAR-3, and SK-OV-3	Inhibited prosurvival signaling proteins Akt, mTOR and NF-kB; inhibited NF-κB-regulated anti-apoptotic proteins Bcl-2, Bcl-xL, c-IAPl and survivin	(Gao et al., 2014)
Osteosarcoma	MNNG and 143B	Decreased expression of Akt, mTOR, and NF-κB	(Mori et al., 2017)
Oral cancer	KBv200	Decreased P-gp through interrupt protein stability in MAPK and PI3K/Akt pathways	(Yan et al., 2017)
	CAL-27 and SCC-25	G1 phase arrest and MAPK/Erk1/2 and Akt signaling inhibition	(Wu et al., 2019)
ESCC	EC9706, EC109, and KYSE30	Inhibited NF-κB pathway, synergistic effect with 5-FU	(Tu et al., 2018)

ESCC, esophageal squamous cell carcinoma; ROS, reactive oxygen species.

## Data Availability

All datasets analyzed for this study are included in the manuscript and the supplementary files.

## Author Contributions

JZ and HC conceived this review; JL and YY wrote the article. HS, YL, and CS revised the article.

## Funding

The work was supported by National Natural Science Foundation of China (81473320 and 81773888), Fund of Guangdong Science and Technology Department (2016A020226024), Fund of Guangzhou Science and Technology Program (201707010048), Fund of Guangdong Education Department (2015KTSCX112), and Fund of Construction of High Level Universities in Guangdong (Nanshan Scholars Program and Academic Backbone Program).

## Conflict of Interest Statement

HS was employed by Infinitus (China) Company Ltd.

The remaining authors declare that the research was conducted in the absence of any commercial or financial relationships that could be construed as a potential conflict of interest.
